# People with dementia in nursing home research: a methodological review of the definition and identification of the study population

**DOI:** 10.1186/s12877-016-0249-7

**Published:** 2016-04-05

**Authors:** Rebecca Palm, Saskia Jünger, Sven Reuther, Christian G. G. Schwab, Martin N. Dichter, Bernhard Holle, Margareta Halek

**Affiliations:** German Centre for Neurodegenerative Diseases (DZNE), Site Witten, Stockumer Str. 12, 58453 Witten, Germany; Faculty of Health, School of Nursing Science, Witten/Herdecke University (UW/H), Stockumer Str. 12, 58453 Witten, Germany; Hannover Medical School, Institute of General Medicine, Carl-Neuberg-Str. 1, 30625 Hannover, Germany

**Keywords:** Dementia, Cognitive impairment, Diagnosis, Symptom assessment, Nursing home, Health services research

## Abstract

**Background:**

There are various definitions and diagnostic criteria for dementia, leading to discrepancies in case ascertainment in both clinical practice and research. We reviewed the different definitions, approaches and measurements used to operationalize dementia in health care studies in German nursing homes with the aim of discussing the implications of different approaches.

**Methods:**

We conducted a systematic search of the MEDLINE and CINAHL databases to identify pre-2016 studies conducted in German nursing homes that focused on residents with dementia or cognitive impairment. In- or exclusion of studies were consented by all authors; data extraction was independently carried out by 2 authors (RP, SJ). The studies’ sampling methods were compared with respect to their inclusion criteria, assessment tools and methods used to identify the study population.

**Results:**

We summarized case ascertainment methods from 64 studies. Study participants were identified based on a diagnosis that was evaluated during the study, or a recorded medical dementia diagnosis, or a recorded medical diagnosis either with additional cognitive screenings or using screening tests exclusively. The descriptions of the diagnostics that were applied to assess a diagnosis of dementia were not fully transparent in most of the studies with respect to either a clear reference definition of dementia or applied diagnostic criteria. If reported, various neuropsychological tests were used, mostly without a clear rationale for their selection.

**Conclusion:**

Pragmatic considerations often determine the sampling strategy; they also may explain the variances we detected in the different studies. Variations in sampling methods impede the comparability of study results. There is a need to consent case ascertainment strategies in dementia studies in health service research in nursing homes. These strategies should consider resource constraints and ethical issues that are related to the vulnerable population of nursing home residents. Additionally, reporting about dementia studies in nursing homes need to be improved. If a diagnosis cannot be evaluated based on either ICD or DSM criteria, the study population may not be reported as having dementia. If a diagnosis is evaluated based on ICD or DSM criteria within the study, there is a need for more transparency of the diagnostic process.

**Electronic supplementary material:**

The online version of this article (doi:10.1186/s12877-016-0249-7) contains supplementary material, which is available to authorized users.

## Background

Health care service research aims to evaluate care strategies by obtaining an understanding of the causal factors in improving dementia-related outcomes for individuals living in nursing homes [1]. It is self-evident that a valid method to ascertain a case is a prerequisite for any study [2]. Case ascertainment strategies must rely on an established definition, and diagnostic criteria must be both valid and feasible to conduct.

Dementia may be defined as a clinical syndrome of mental capacity characterized by a substantial global decline in cognitive function that is not attributable to altered consciousness; it consists of a combination of symptoms attributable to various causes or pathological events [3]. Following the Diagnostic and Statistical Manual (DSM) 5th edition [4], dementia is subsumed under the entity of neurocognitive disorders (NCD). The diagnostic criteria for NCD are as follows:

i. Evidence of a significant cognitive decline from a previous level of performance in one or more cognitive domains (complex attention, executive function, learning and memory, language, perceptual-motor or social cognition)
ii. The cognitive deficits interfere with independence in everyday activities (present in major NCD but not in mild NCD)
iii. The cognitive deficits do not occur exclusively in the context of a delirium
iv. The cognitive deficits are not better explained by another mental disorder

Evidence for the cognitive decline should be based on concerns from the individual patient, a knowledgeable informant, or the clinician regarding the presence of a significant decline in cognitive function and a substantial impairment in cognitive performance; ideally, this evidence will be documented by standardized neuropsychological testing. The DSM-V manual notes that both concern and objective evidence are required to establish a diagnosis because they are complementary. If alterations in cognition are only tested utilizing objective measures, a disorder may be underdiagnosed in individuals who demonstrate high-functioning performance and score in the “normal” range but are still substantially impaired relative to their baseline performance. On the other hand, an exclusive reliance on subjective symptoms may result in underdiagnosing individuals who deny or fail to express their impairments. In either case, it is essential to interpret results in comparison with the individual patients’ prior performance.

The International Classification of Diseases (ICD) defines dementia as a decline in both memory and other cognitive abilities such as deterioration in judgment and thinking or the general processing of information. The decline in memory and cognitive abilities should be objectively verified by obtaining a reliable history from an informant and supplemented by neuropsychological tests or quantified cognitive assessments. The diagnosis of mild impairment is based on the degree of memory loss that interferes with everyday activities. A diagnosis of dementia requires, in addition to the previously mentioned symptoms for mild impairment, a decline in emotional control or motivation, changes in social behavior, and the absence of delirium. Furthermore, the described symptoms must be present for at least 6 months [5].

The diagnostic process follows two steps, starting with the initial recognition of the dementia syndrome and ending with the specification of an etiological subtype. The diagnostic criteria for each of the subtypes are specified in the DSM-V, ICD-10 [4, 5] and publications from different disease-related societies or national institutes (e.g., the National Institute of Neurological and Communicative Disorders and Stroke, NINCDS) [6–10]. The subtype differentiation may require biomarker diagnostics such as genetic, blood and liquor testing or diverse imaging procedures.

For more than 3 decades, the history of classification systems for mental disorders has reflected both many changes and a relative emphasis on phenomenology, etiology, and course as defining features. When the DSM-III’s (1980) explicit diagnostic criteria were introduced (Feighner-criteria), several revisions followed: the DSM-III-R (revision) in 1987, the DSM-IV in 1994, and the DSM-V in 2013. The ICD introduced diagnostic criteria in its revised version 9, ICD-9-CM (for clinical modification). Every revision aimed both to reduce inconsistencies and to improve clarity and precision. Different versions included changes in the classification, diagnostic criteria sets, and descriptive texts [11].

Dementia diagnoses pose several challenges. First, the lack of valid biomarkers forces healthcare providers to diagnose dementia based on the presence of specific symptoms and the elimination of other conditions with similar symptoms. To arrive at a clinical diagnosis of dementia, a combination of basic assessments,—including physical, psychopathological and basic neuropsychological examinations—are recommended [12, 13]. For the basic neuropsychological diagnosis, different short screening tests such as the Mini Mental Status Examination (MMSE) [14, 15], the DemTect [16] and the Clock Drawing Test [17] are recommended. In clinical practice, these tests should be administered to every patient with dementia or suspected dementia on a regular basis both to quantify cognitive impairments and to enable providers to supervise the progression of the disease. Comprehensive neuropsychological diagnostics are necessary either if diagnostic findings are not congruent in the early stage of dementia or if an etiological classification is required. A dementia diagnosis should not be based solely on one neuropsychological test but instead must be based on both behavioral symptoms and impairment assessments related to daily function. Additionally, blood tests and imaging diagnostics should be conducted to assure both the diagnosis and its etiology [18].

The reliability, validity and utility of the new diagnostic criteria have discussed by clinicians and researchers since the date of their release; furthermore, a consensus about how to best diagnose dementia has still not been found [19, 20]. One result of this controversy is the methodological differences in epidemiological studies that lead to discrepancies in case ascertainment and varying incidence and prevalence rates [3, 21]. Another limitation of the generalizability of study results on dementia in nursing homes originates from the methodological difficulties of assessing the disease in the oldest old population. The normative data necessary to determine “impairment” for this population are lacking and therefore, aging is constitutionally associated with a decline in both cognition and function. Consequently, pathological processes are difficult to distinguish from normal aging; in addition, alterations in function and sensory impairments may both affect subjects’ cognitive performance and limit an accurate assessment [20].

The quality of a dementia diagnosis in German nursing homes is suboptimal: many studies of nursing home residents show both vast inaccuracies and diagnostics that do not conform to either the ICD or the DSM. Studies from Germany show that between 30 and 40 % of people with dementia living in nursing homes are not accurately diagnosed; for these residents, either an etiological differentiation is missing or there is an inappropriate diagnosis [22–24]. Inaccurate diagnosis has also been found in studies from the United States, Norway, Israel and Ireland [25–29].

Nursing home research is challenged by inaccurate recorded diagnoses. Ignoring inconsistencies in a recorded dementia diagnosis may provoke misclassifications, bias selection and confound study results [30]. However, the evaluation of a diagnosis within a study is resource-intensive and may be ethically questionable because the diagnosis process is reported as burdensome and stigmatizing [31, 32].

Researchers must develop a sampling strategy that eliminates inconsistencies while remaining both reliable and practicable. We assume that in the past, researchers defined dementia differently and applied different methods to identify residents with dementia in nursing homes. Therefore, we investigated the methods used to define dementia and to identify people with dementia in health services research. Although we assume that the delineated problem is obvious in various countries, we limited the scope of our study to research undertaken in a single country (Germany). In Germany, physicians are not constantly present in nursing homes, and nurses are not allowed to assist in diagnostic procedures; resources for dementia diagnostics in primary care are scarce [33]; hence, diagnostics are often superficial, performed rapidly and lacking recommended measures [34, 35]. Due to the free choice of medical practitioners that is guaranteed by law in Germany, residents within one nursing home may be cared for by different practitioners, making it difficult for researchers to reconstruct the diagnostic procedures that were applied by various medical doctors. These conditions require a carefully considered sampling strategy. The narrowed focus on one country ensures the comparability of the studies because the nursing-home conditions are identical. This allows a synthesis and comparison of the various methods used in the included studies.

Because cognitive impairment (CI) is the leading symptom in dementia and the conditions “dementia” and “CI” are sometimes used interchangeably in scientific publications, the scope of our review encompasses studies that do not exclusively focus on residents with dementia but instead include residents with CI. Provided we found differences, we discuss the implications of different methods on the generalizability and comparability of the study findings.

The following research questions were addressed:

- *How is dementia defined and measured in health services research studies in German nursing homes?*
- *Which implications can be derived for health services research in nursing homes?*

Based on the results, we discuss the implications of the different methods and suggest principles to guide future studies.

## Methodology of the review

Compared to systematic reviews that aim to answer questions about the effectiveness of clinical procedures, there are few well-defined tools and processes for methodological reviews; not all items of the Preferred Reporting Items for Systematic Reviews and Meta-Analyses (PRISMA) guidelines are applicable (see PRISMA checklist in the Additional file 1: Table S1) [36]. Unlike a traditional Cochrane-style review, Lilford and colleagues [36] recommend methodological reviewers not to obtain a thorough collection of studies, but to ensure a wide search that provides an overview of the topic. They also recommend various safeguards to reduce potential bias, such as multi-disciplinary teams and peer reviewing of the final report. The methods applied in this review consider these recommendations.

### Literature search strategy

We conducted a systematic literature search to identify relevant publications in the MEDLINE [PubMed] and CINAHL [EBSCO] databases in January 2014 (MND) and updated it in December 2015 (RP).These databases were chosen because we considered them as the most relevant to the field of health services research. We detail the search strategy in the Additional file 2: Table S2 and Table S3. The search was not limited to a specific time and study design, but it was limited to the German and English languages. After the initial title and abstract screening, publications were excluded if they were considered irrelevant to the topic or setting (RP, CGGS). The remaining publications were divided to be read in full-text and to be excluded according to predefined criteria by one research team member (RP, SR, MND, CGGS, BH, MH). Publications fulfilling the following criteria were included for review: the research aim or question focused on residents with dementia or CI in German nursing homes and was answered based on empirical data. Each decision to exclude a publication was discussed and consented by the research team.

### Data extraction and method of analysis

Two researchers (RP, SJ) read the included articles in full-text and extracted the data: SJ conducted an initial data extraction, and RP checked all of the information for accuracy. In the first step of the data extraction, an overview of the data on the study aim, design and sample size were compiled. In the second step, the publications were sorted according to sample determination procedure. Publications from one study using the same method to identify residents with dementia/CI were summarized; this was the case with publications from large projects with various research aims and questions. To answer the research questions, the following information was extracted: information on the method of sample determination, the definition and criteria used for dementia diagnostics, the screening instrument(s) used, and the qualification and training of the professionals involved. The extracted findings and key characteristics of each method were summarized in an overview, and differing approaches were compared.

## Results

**Fig. 1:**
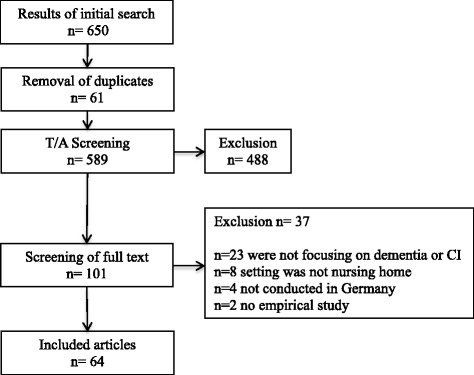
Flow chart of literature search

The literature search yielded 650 articles. Sixty-four articles were identified to answer our research questions. The results of the literature search can be seen in the flow chart in Fig. 1.

The characteristics of the included studies are presented in Table 1.

**Table 1:** Characteristics of included studies

Publication	Author	Study aim	Study design	Sample size	
				NH	P
	Experimental studies				
[54]	Bär et al. 2006	Efficacy of an individual approach in the care of people with dementia to stimulate positive emotions	CT	N/A	46
[82]	Berg et al. 2010	Efficacy of snoezelen, structured reminiscence therapy and 10-minute activation on apathy in dementia	C-RCT	N/A	360
[107]	Dichter et al. 2015	Testing the effectiveness of Dementia Care Mapping on PwD and caregivers, exploring implementation facilitators and barriers	CT	9	154
[76]	Graessel et al. 2011	Efficacy of a non-pharmacological intervention on the cognition of residents with dementia	RCT (Follow-up)	5	98
[108]	Halek et al. 2013	Efficacy of dementia care mapping on the quality of life of residents with dementia	CT	9	N/A
[70]	Kuske et al. 2009	Effectiveness of a nursing home training staff for the interaction between residents with dementia and their caregivers	C-RCT	6	298
[77]	Luttenberger et al. 2012	Efficacy of a non-pharmacological intervention on dementia symptoms and need of care in NH residents with dementia	RCT	5	139
[78]	Luttenberger et al. 2012	Sustainability of a non-pharmacological intervention after 10 months	RCT (Follow-up)	5	61
[48]	Majic et al. 2013	Efficacy of animal-assisted therapy on agitation/aggression and depression in nursing home residents with dementia	RCT	18	65
[80]	Pickel et al. 2011	Efficacy of occupational group therapy in dementia	CT	N/A	56
[51]	Rapp et al. 2013	Efficacy of a complex guideline-based intervention on agitation and the use of psychotropic drugs	C-RCT	18	304
[47]	Reuther et al. 2014	Effect evaluation of dementia-specific case conferences (study protocol)	C-RCT	12	360
[109]	Schäufele et al. 2013	Efficacy of an interdisciplinary guideline to enhance the mobility of residents with dementia in nursing homes	CT	31	707
[110]	Schäufele et al. 2015				
[81]	Treusch et al. 2015	Effect evaluation of an occupational and sports therapy intervention for PwD in NH	C-RCT	18	117
	Observational studies				
[85]^a^	Afram et al. 2014	Exploration of reasons and variations for institutionalization of people with dementia in 8 European countries according to caregivers	Cross-sectional	3	786
[86]^a^	Alvira et al. 2015	Description of the association between reactions of informal caregivers of people with dementia and health outcomes from 8 European countries	Cross-sectional	N/A	119
[61]	Becker et al. 2005	Development and validation of an instrument to assess quality of life in dementia	Cross-sectional	11	121
[87]^a^	Beerens et al. 2014	Exploration of the variance of quality of life and quality of care for people with dementia from 8 European countries	Cross-sectional	N/A	119
[88]	Beerens et al. 2015	Assessment of factors that contribute to the change of quality of life of people with dementia recently admitted to an NH from 8 European countries	Longitudinal	N/A	791
[22]	Brune-Cohrs et al. 2007	Quality of dementia diagnosis in nursing homes	Cross-sectional	2	200
[89]^a^	De Mauleon et al. 2014	Determination of factors associated with the antipsychotic prescription for PwD in 8 European countries	Cross-sectional	N/A	119
[83]	Dettbarn-Reggentin 2005	Evaluation of milieu-therapeutic living units on residents with dementia	Longitudinal	3	60
[53]	Dichter et al. 2013	Validation of the QUALIDEM in nursing homes	Cross-sectional	43	634
[111]	Dichter et al. 2014	Testing of the inter- and intra-rater reliability of the QUALIDEM instrument to measure quality of life in PwD	Cross-sectional	9	161
[44]^a^	Foebel et al. 2014	Description of patterns of antipsychotic drug use in PwD in nursing homes in 7 European countries and Israel	Cross-sectional	9	496
[72]	Geiger-Kabsich and Weyerer 1993	Validity of the Alters-Konzentrations-Test	Cross-sectional	2	71
[91]	Gietzelt et al. 2014	Study protocol for an RCT testing the effectiveness of behavioral treatment for mild Alzheimer’s patients	Longitudinal	1	40
[41]	Graessel et al. 2009	Validation of the Erlangen Test of Activities of Daily Living (E-ADL)	Cross-sectional	2	46
[45]	Gräske et al. 2014	Examination of variability and associated factors of quality of life ratings	Cross-sectional	5	133
[66]	Jakob et al. 2002	Prevalence and incidence of dementia in nursing homes compared to private households	Longitudinal	N/A	192
[69]	Köhler et al. 2007	Validation of the Dementia Screening Scale (DSS)	Cross-sectional	20	589
[37]	Kölzsch et al. 2012	Description of pain treatment in nursing home residents with CI	Cross-sectional	40	560
[68]	König et al. 2014	Comparison of the costs of care for community-dwelling PwD and PwD living in nursing homes	Cross-sectional	N/A	48
[62]	Lueken et al. 2007	Development of a short version of the Apathy Evaluation Scale specifically adapted for nursing home residents with dementia	Cross-sectional	N/A	356
[79]	Luttenberger et al. 2012	Revalidation of the E-ADL scale	Cross-sectional	5	139
[49]	Majic et al. 2010	Pharmacotherapy in residents with dementia	Cross-sectional	18	304
[50]	Majic et al. 2012	Correlation of agitation and depression in nursing home residents with dementia	Cross-sectional	18	304
[46]	Makai et al. 2014	Validation of the ICECAP-O measure for wellbeing in older PwD in NH and exploration of response-associated factors	Cross-sectional	1	95
[58]	Marquard and Schmieg 2009	Relationship between architectural characteristics of the nursing home and the residents ability to perform way finding tasks	Cross-sectional	N/A	450
[59]					
[75]	Meyer-König et al. 1984	Examination of nursing home residents with a chronic brain syndrome	Cross-sectional	N/A	163
[38]	Osterbrink et al. 2012	Prevalence of pain in nursing home residents with various cognitive functions	Cross-sectional	13	436
[57]	Palm et al. 2013	Evaluation of the provision of dementia care and identification of resident- and facility-related factors associated with quality of life and behavior	Longitudinal	N/A	N/A
[52]	Palm et al. 2015	Comparison of case conferences between dementia-specialized versus traditional care units	Cross-sectional	51	888
[67]	Riedel et al. 2013	Prevalence of Parkinson’s disease, associated dementia and depression in Dresden	Cross-sectional	36	195
[24]	Schäufele et al. 2013	Prevalence of dementia and provision of dementia care in nursing homes	Cross-sectional	58	4481
[65]	Schuler et al. 2007	Validation study of the “Pain Assessment in Advanced Dementia Scale” (PAINAD-G) in nursing home residents	Cross-sectional	8	99
[39]	Schumacher et al. 1997	Prevalence of depression and CI in nursing home residents	Cross-sectional	3	380
[23]	Seidl et al. 2007	Prevalence of non-cognitive symptoms and psychopharmacological treatment in nursing home residents with dementia	Cross-sectional	N/A	145
[64]	Seidl et al. 2009	Comparison of neurological soft signs of residents with AD with residents without cognitive impairments	Cross-sectional	N/A	120
[63]	Seidl et al. 2011	Description of autobiographical memory deficits in residents with dementia	Cross-sectional	N/A	239
[84]	Theison et al. 2009	Association of agitation in the morning and depression	Cross-sectional	3	110
[73]	Weyerer et al. 1990	Validation of the Brief-Assessment-Interview	Cross-sectional	1	32
[71]	Weyerer et al. 1995	Prevalence of dementia and depression in nursing home residents from Mannheim and Camden	Cross-sectional	12	542
[56]	Weyerer et al. 2004	Comparison of residents from day-care centers and nursing homes	Cross-sectional	47	1644
[42]	Weyerer et al. 2005	Evaluation of special and traditional dementia care in nursing homes	Cross-sectional	31	1644
[43]	Weyerer et al. 2010	Evaluation of special and traditional dementia care in nursing homes	Cross-sectional	31	1644
[90]	Wubker et al. 2015	Comparison of costs for PwD receiving home care versus nursing home care in 8 European countries	Cross-sectional	N/A	76
[40]	Wulff et al. 2012	Description of perceived autonomy of nursing home residents with and without CI	Cross-sectional	40	560
[93]	Zenthofer et al. 2014	Comparison of oral hygiene and health status of nursing home residents with and without dementia.	Cross-sectional	N/A	93
	Qualitative studies				
[55]	Bär et al. 2003	Identification of characteristic situations accompanied by positive emotions	Qualitative	N/A	29
[60]	Becker et al. 2006	Identification and cross-validation of patterns of competence in nursing home residents.	Qualitative	N/A	362
[92]	Nordheim et al. 2015	Evaluation of the use of tablet PCs in PwD in NH	Qualitative	1	14

*AD* Alzheimer’s disease, *C-RCT* Cluster-randomized controlled trial, *CT* controlled trial, *N/A* not available, *NHs* nursing homes, *P* Participants, *PwD* People with dementia, *RCT* Randomized controlled trial

^a^The number of participants reported from this international study refers to the German sample only

### Methods to identify the study population residents with dementia

All 64 articles focused on the population of nursing home residents with CI. In 60 articles, dementia was specified as the etiology of CI. In these articles, the term “dementia” was used consistently in the title, abstract, key words and research questions. Four articles focused their research question on residents with CI without any specification of etiology, which means they did not clearly define dementia residents as their study population [37–40]. However, in these articles the terminology was not consistent throughout the article: the term dementia and CI were used interchangeably.

The articles focusing on residents with dementia used different methods to determine their study sample. We identified 4 methods that were clearly described.

Nursing home residents with dementia were identified in one of the following ways:

1. A study diagnosis that was evaluated during the study.
2. A recorded medical diagnosis.
3. A recorded medical diagnosis and an additional cognitive screening performed during the study.
4. A cognitive or accordant screening.

1. Study diagnosis
  In 17 articles, a study diagnosis of dementia was used to determine the participant sample (Table 2). The diagnosis was newly assessed either in all residents or in residents with a diagnosis that had previously been documented.

**Table 2:** Overview of studies that determine study participants with dementia based on a study diagnosis

Publications	Method of sample determination	Definition and diagnostic criteria used for (new) dementia diagnosis	Screenings performed	Qualification and training of professionals performing screenings/diagnostics
[22]	Diagnostics performed for every resident with an existing dementia diagnosis	**▪** Clinical examination **▪** Semi-standardized interview and neuropsychological testing according to ICD-10 criteria **▪** Consolidation of existing diagnostic findings **▪** NINCDS-ADRDA criteria for diagnosis of AD **▪** Consensus criteria for frontotemporal dementia **▪** Petersen criteria for mild cognitive impairment (MCI)^a^ **▪** NINDS-AIREN for vascular dementia	MMSE, CDR, Behave-AD, BPRS, HDRS 17, B-ADL	Diagnosis: Physician with experience in geriatric psychiatry
[23]	Diagnostics performed by physicians from the research team for every resident fulfilling one of the criteria: **▪** Presence of dementia diagnosis in the nursing records **▪** Resident appears forgetful **▪** Resident has problems with orientation within the NH	**▪** NINCDS-ADRDA criteria on the basis of clinical examination, existing assessments of status and progress, existing diagnostic findings (technical investigations) **▪** Dementia was classified into different types: AD, vascular dementia, mixed type, frontotemporal dementia	MMSE, GDS, Clock Drawing Test, CERAD entire battery, BAGI, AES, NPI	Screening instruments and diagnosis: Experienced geriatric psychiatrist with formal training in the administration and scoring of the respective instruments.
[60]				
[61]				
[62]				
[63]				
[64]				
[65]				
[66]	Diagnostics performed for a random sample of nursing home residents	**▪** SIDAM-interview for the assessment of cognitive function; in case of severe physical impairment CDR **▪** Diagnosis of etiological subtype based on the findings from the SIDAM-interview **▪** Diagnosis discussed in an expert conference of physicians and psychologists according to DSM-III-R	SIDAM, MMSE or CDR	Diagnosis: Physicians and psychologist who received training in conducting structured interviews
[67]	Diagnostics performed for a random sample of residents with Parkinson’s disease	**▪** Diagnosis assessed according to DSM-IV-TR criteria using the SIDAM-interview, clinical examination, medical history	SIDAM, MMSE, PANDA (subsample)	Screening instruments and diagnosis: Study monitor with a medical education
[69]	Diagnostics in the study was performed for all nursing home residents.	**▪** No definition or diagnostic criteria stated **▪** Diagnosis assessed using the CDR (≥ 1)	MMSE, BAS-DEM, CDR, DSS, BAI	Diagnosis: Trained clinical psychologist Screening (DSS): Licensed geriatric nurses with frequent contact with the residents during the previous 4 weeks
[70]	Diagnostics in the study was performed for every consenting resident.	**▪** No definition or diagnostic criteria stated **▪** Diagnosis assessed using the CDR (≥ 1)	CDR, MMSE, Barthel-Index	Diagnosis: Determined in multidisciplinary consensus conferences held by psychiatrists, clinical psychologists and health and nursing specialists. Screening instruments: Not specified
[71]	Diagnostics performed for all NH residents	**▪** No definition or diagnostic criteria stated **▪** Diagnosis assessed using the BAI (3-8 = mild to severe dementia)	BAI	Interviews performed by trained NH staff with experience in clinical psychology and psychiatry
[72]	Diagnostics performed for NH residents able to be interviewed	**▪** Assessment of diagnosis according to Feighner-criteria **▪** Dementia severity cutoff value (MMSE ≤ 23 minimum mild dementia)	AKT, BAI,	Diagnosis: NH manager experienced in psychiatry
[73]	Diagnostics in the study performed for a non-defined sample of NH residents	**▪** Diagnosis assessed according to the Feighner criteria and compared with a diagnosis assessed with the BAI (BAI 0-2 = most likely no dementia, 3-7 = mild to moderate dementia, 8 = severe dementia)	BAI	Diagnosis (Feighner criteria): experienced NH manager
[75]	Diagnostics for organic psycho syndrome (OPS) (dementia) performed in a non-defined sample of NH residents	**▪** Differentiation of OPS severity based on an assessment of cerebral dysfunction and changes in personality	Not specified	Not specified
[68]	Diagnostics in the study performed for all included participants	**▪** SIDAM interview was conducted **▪** Diagnosis was based on a consensus between study interviewers and an experienced geriatrician or geriatric psychiatrist according to DSM IV for Alzheimer or ICD-10 or DSM III R criteria for multi-infarct dementia and other etiology **▪** Diagnostic criteria: objective deficits in memory and another cognitive domain, impairment in activities of daily living **▪** Classification of dementia was based on the CDR (≤ 1 = mild, 2 = moderate, 3 = severe) **▪** Assessed data were combined into simple and weighted count scores	SIDAM, CDR, MMSE, Barthel-Index for ADL impairment, IADL impairment scale, 28 chronic conditions	Trained physicians or psychologists conducted interviews with participants and their caregivers.

*AD* Alzheimer’s disease, *ADL* Activites of daily living, *AES* Apathy Evaluation Scale, *AKT* Alters-Konzentrationstest, *B-ADL*, Bayer-Activities of Daily Living Scale, *BAGI* Bielefelder Autobiografisches Gedächtnisinventar, *BAI* Brief Assessment Interview, *BAS-Dem* Brief Assessment Schedule, *Behave-AD* Behavioral Pathology in Alzheimer’s Disease, *BPRS* Brief Psychiatric Rating Scale, *CDR* Clinical Dementia Rating, *CERAD* The Consortium to Establish a Registry for Alzheimer’s Disease, *DSS* Dementia Screening Scale, *DSM* Diagnostic Statistical Manual, *E-ADL* Erlangen Test for Activities of Daily Living, *GDS* Global Deterioration Scale, *HDRS 17* Hamilton Depression Scale 17, *IADL* Instrumental Activities of daily living, *MMSE* Mini Mental State Examination, *NOSGER* Nurses’ Observation Scale, *NPI* Neuropsychiatric Inventory, *PANDA* Parkinson Neuropsychometric Dementia Assessment, *SIDAM* Structured Interview for the Diagnosis of Alzheimer’s Dementia, Multi-infarct dementia and dementias of other etiology

^a^Mild Cognitive Impairment is defined as a cognitive disorder that is characterized by impaired memory function and learning abilities. None of the symptoms are severe enough to justify a dementia diagnosis [5]
2. Recorded medical diagnosis
  In 6 articles, the recorded diagnosis was the criterion for determining the sample participants (Table 3). In the study by Graessel et al. [41], attending physicians confirmed the recorded diagnosis; in the study by Weyerer et al. [42, 43], the diagnosis was used as an indirect inclusion criterion. In this study, the dementia diagnosis was an admission criterion for the care unit that participated in the study (Dementia Special Care Unit). This study does not report whether the diagnosis was confirmed. In 3 articles, the diagnosis was obtained from the residents’ records [44–46]; 1 article reported that the dementia type was evaluated according to the ICD classification (as recorded), and the dementia severity evaluation was guideline-based and performed according to the recommended MMSE cutoff values [46].

**Table 3:** Overview of studies that defined study participants with dementia based on a recorded diagnosis

Publications	Method of sample determination	Definition and diagnostic criteria used for (new) dementia diagnosis	Screenings performed	Qualification and training of professionals performing screenings/diagnostics
[41]	A suspected dementia diagnosis (by nursing staff) or existing dementia diagnosis confirmed by the attending GP	**▪** ICD-10 criteria **▪** Dementia severity stages: MMSE: 0-9 severe, 10-17 moderate, ≥ 18 mild dementia	MMSE, GDS, NOSGER, E-ADL,	Not specified
[42]	Diagnostics not performed in the study, but the admission criteria for the living unit were used as inclusion criteria (dementia diagnosis, minimum of care level 2, behavioral problems according to the CMAI, and mobility)	**▪** No information given on the diagnostic procedure of the existing diagnosis	DSS	Professional nursing staff, training of raters is not specified
[43]				
[44]	Dementia diagnosis was derived from the interRAI LTCF assessment in the records	**▪** No information given on the diagnostic procedure of the existing diagnosis	InterRAI (LTCF)	Not specified
[45]	Residents with a medical diagnosis of dementia were included.	**▪** No information given on the diagnostic procedure of the existing diagnosis	GDS	Measures were assessed by nurses.
[46]	Residents with a medical diagnosis of dementia were included.	**▪** Assessment of the dementia type according to the ICD-10 classification (as recorded) **▪** Assessment of dementia severity according to the German guideline for dementia and recommended MMSE cutoff values (0-9 severe, 10-19 moderate, 20-26 mild)	MMSE	Not specified.

*DSS* Dementia Screening Scale, *E-ADL* Erlangen Activities of Daily Living, *GDS* Global Deterioration Scale, *interRAI LTCF* international Resident Assessment Instrument Long Term Care Facility, *MMSE* Mini Mental State Examination, *NOSGER* Nurses’ Observation Scale
3. Recorded medical diagnosis and screening
  In 20 articles, the recorded medical diagnosis of dementia was used as a criterion to determine the sample participants, but it was combined with the results of a cognitive screening measure (Table 4). In 19 studies, a resident was included if the result of the MMSE also indicated CI; 1 study included a resident if a diagnosis was recorded and the result of a screening using the Functional Assessment Staging Test (FAST) indicated dementia [47]. One study used a stepwise approach [48–51]: in the case of an incongruity between the diagnosis and the MMSE result, additional diagnostics were performed to decide whether a resident fulfilled the inclusion criteria.

**Table 4:** Overview of studies that defined study participants with dementia based on a recorded diagnosis and additional cognitive screenings

Publications	Method of sample determination	Definition and criteria used for (existing) dementia diagnostics	Screenings performed to define and describe dementia	Qualification and training of professionals involved
[48]	Residents with dementia were identified using a mixed stepwise approach: 1st step: Multiple combined inclusion criteria: ▪ Presence of dementia diagnosis in the nursing and medical records and ▪ MMSE ≤ 24 2^nd^ step: In case of incongruity of diagnosis and MMSE result, dementia diagnostics were performed, diagnostics were also performed for residents with a suspected dementia but no diagnosis ▪ Exclusion criteria: Presence of other neurological/psychiatric diseases that could explain patients’ decline in cognitive function (schizophrenia, bipolar disorders, mental retardation)	▪ Existing dementia diagnosis performed in 70 % by GPs and 30 % by medical specialists and according to ICD-10 criteria ▪ An ICD-10/DSM-IV conform study diagnosis was assessed based on clinical investigation and MMSE ▪ Dementia severity stages: MMSE: 0-9 severe/very severe; 10-18 moderate; 19-24 = mild dementia	MMSE, FAST, AES, NPI	Diagnosis: Physician from the research team who was experienced in geriatric psychiatry Screening instruments: Specifically trained raters, including medical students with an advanced academic degree and physicians experienced in geriatric psychiatry
[49]				
[50]^a^				
[51]				
[81]^b^				
[76]	Residents with dementia were identified using two combined inclusion criteria: ▪ Confirmed presence of primary degenerative dementia and ▪ MMSE < 24 ▪ Exclusion criteria: presence of other neurological/psychiatric diseases that could explain patients’ decline in cognitive function (such as addiction, major depression, or schizophrenia), high nursing care needs, blindness, deafness	▪ Dementia diagnosis confirmed according to ICD-10 (F00, F03, or G30), exclusion of vascular (F01) and secondary (F02) dementia ▪ Dementia severity stages: MMSE: 0-9 severe; 10-17 moderate; 18-23 mild dementia	MMSE, ADAS (cognitive subscale), NOSGER (subscale mood), E-ADL	Diagnosis: confirmed by the attending physician Screening instruments: Psychology students in their final year who had received training
[77]				
[78]				
[79]				
[80]	Residents with dementia were identified using two combined inclusion criteria: ▪ Presence of a dementia diagnosis; and ▪ MMSE < 24, GDS stadium 4, 5 or 6 ▪ Exclusion criteria: presence of other neurological/psychiatric diseases that could explain patients’ decline in cognitive function (such as addiction, major depression, or schizophrenia)	Dementia diagnosis according to ICD-10 in the doctoral records	MMSE, GDS, ADAS (cognitive subscale), NOSGER (subscale mood), E-ADL	Not specified
[82]	Residents with dementia were identified using 2 combined inclusion criteria: ▪ Existing diagnosis of dementia and ▪ MMSE ≤ 24 ▪ Exclusion criteria: Korsakoff’s syndrome or CI caused by diseases other than dementia	No specified information given on the diagnostic procedure of the existing diagnosis	MMSE, CDR, AES	Not specified
[83]	Residents with dementia were identified using 2 combined inclusion criteria: ▪ Admission criteria to the living unit were used as inclusion criteria (presence of a dementia diagnosis) and ▪ MMSE < 18	No specified information given on the diagnostic procedure of the existing diagnosis	MMSE, NOSGER, GDS	Not specified
[84]	Residents with dementia were identified using two combined criteria: ▪ Presence of a dementia diagnosis; and ▪ MMSE ≤ 27 and DemTect (scores of 6-8) for residents with a MMSE score between 24-27	▪ No specified information given on the diagnostic procedure of the existing diagnosis ▪ Dementia severity stages: MMSE: ≤ 10 severely demented; 11-19 moderately demented, 20-27 mild dementia	MMSE, DemTect	Not specified
[85]	Residents with dementia were identified using 2 combined criteria: ▪ S-MMSE-Score ≤ 24 ▪ Exclusion criteria: primary psychiatric diagnosis or Korsakov’s syndrome	▪ No specified information given on the diagnostic procedure of the existing diagnosis ▪ Different dementia severity stages were used	MMSE, NPI, Katz-Index	Formal diagnosis of dementia as determined by a healthcare professional (physician, psychiatrist, neurologist, geriatrician, general practitioner)
[86]				
[87]				
[88]				
[89]				
[90]				
[47] (Study Protocol)	Residents with dementia were identified using 2 combined criteria. ▪ Medical diagnosis of dementia ▪ FAST > 1	▪ No specified information given on the diagnostic procedure of the existing Diagnosis	FAST, NPI, PSMS	▪ Data were assessed by trained study assistants who interview two caregivers simultaneously ▪ Study assistants (mainly students) undergo a 2-day training on the use of the questionnaires and receive a manual ▪ The first data collection was assisted by senior and junior researchers

*AD* Alzheimer’s disease, *ADAS* Alzheimer’s disease Assessment Scale, *AES* Apathy Evaluation Scale, *CDR* Clinical Dementia Rating, *E-ADL* Erlangen Test for Activities of Daily Living, *FAST* Functional Assessment Staging, *GDS* Global Deterioration Scale, *MMSE* Mini Mental State Examination, *NOSGER* Nurses’ Observation Scale, *NPI* Neuropsychiatric Inventory

^a^The reporting of instruments is not consistent across the publications. Therefore, all instruments used were summarized in this group. The studies also differ also regarding the criteria used to diagnose. Majic 2012 referred to DSM- IV, other publications to ICD-10

^b^Reporting of methods and proceedings slightly deviates from that of the other publications originating from this project (validation of recorded dementia diagnosis is not specified; MMSE cutoff 23; no exclusion criteria are reported)
4. Screening
  In 10 articles, residents with dementia were identified by 1 screening or a combination of 2 screenings (Table 5). In these studies, an existing diagnosis was recorded, but it was not used as the inclusion criterion. Palm et al. [52] used the place of residence in a Dementia Special Care Unit as an inclusion criteria as well as the results of a cognitive screening to define their sample. One article reported exclusion criteria that were used to differentiate dementia from other psychiatric disorders [53].

**Table 5:** Overview of studies that defined study participants with dementia based on cognitive screenings

Publications	Method of sample determination	Information about dementia diagnosis	Screenings performed to define and describe dementia	Qualification and training of professionals involved
[108] [53]^a^ [111] [107]	Residents with dementia were identified using one criteria **▪** FAST ≥ 2 **▪** Exclusion criteria: documented diagnosis of schizophrenia or other psychotic disorders	Existing diagnosis of dementia recorded but not used as an inclusion criteria	MMSE^a^, FAST, NPI, PSMS	Screening instruments: Caregivers who were familiar with the resident were interviewed from a trained external research assistant (registered nurses and students in health care study programs)
[52]	Residents were included using 2 criteria: **▪** Place of residence is a Dementia Special Care Unit **▪** Cognitive impairment according to DSS > 2	Existing diagnosis of dementia recorded but not used as an inclusion criteria	DSS, NPI, PSMS	Screening instruments were completed by nurses familiar with the resident; questionnaires were accompanied by a manual to support assessment
[109]^b^ [110]	Residents with dementia were identified using 2 combined criteria **▪** Dementia according to DSS; and **▪** Mobile according to Rivermead Mobility Index	Existing diagnosis of dementia recorded but not used as an inclusion criteria; no information is given on existing diagnosis	DSS, NPI, Barthel-Index	Screening instrument: registered nurses who are familiar with the resident (primary nurse)
[91]	Residents with dementia were identified based on the MMSE screening < 24	No information given on existing diagnosis	MMSE, Barthel-Index	Not specified
[92]	Residents with dementia were identified based on the MMSE screening < 24 **▪** Dementia severity stages: MMSE ≤ 10 severe, 11-17 moderate, ≥ 18 mild	No information given on existing diagnosis	MMSE, Barthel-Index	Not specified
[93]	Residents with dementia were identified based on the MMSE screening **▪** MMSE cutoff ≤ 20	No information given on existing diagnosis	MMSE	MMSE was performed by three psychologists.

*DSS* Dementia Screening Scale, *FAST* Functional Assessment Staging, *MMSE* Mini Mental State Examination, *NOSGER* Nurses’ Observation Scale, *NPI* Neuropsychiatric Inventory, *PANDA* Parkinson Neuropsychometric Dementia Assessment, *PSMS* Physical Self Maintenance Scale

^a^MMSE values are assessed only in a subsample in the study from Dichter et al. (2013) and recalculated into FAST values

^b^The publication is a short report/letter; therefore, we additionally reviewed the official study report for more information
  The 3 studies (4 articles) that investigated residents with CI exclusively used screenings to identify their study participants (Table 6).

**Table 6:** Overview of studies that investigated study participants with cognitive impairment

Publications	Method of sample determination	Screenings performed to define and describe CI	Qualification and training of professionals involved
[37]	Assessment of CI performed for a random sample of NH residents.	MMSE, Barthel-Index	Data were collected through face-to-face interviews by trained research personnel
[40]	**▪** MMSE: 0-17 = severe CI; 18-23 = moderate CI; 24-30 = no or mild CI		
[38]	Assessment of CI was performed for NH residents > 65 years without verbal impairments.	MMSE	Trained study assistants (licensed nurses or students in health care programs) who received a comprehensive training in using the MMSE
	**▪** MMSE: 0-9 severe CI; 10-17 moderate CI; 18-30 = no/mild CI		
[39]	Assessment of CI was performed for all residents of the participating NHs.	MMSE, GDS	Not specified
	**▪** MMSE: < 15 severe CI, 16-20 relevant CI **▪** GDS: 2-3 mild CI, 4-5 moderate CI, 6-7 severe CI		

*CI* cognitive impairment, *GDS* Global Deterioration Scale, *MMSE* Mini Mental State Examination
  Seven publications could not be classified according to the 4 groups explained above (Table 7). In 4 of these publications, all of the residents from a participating nursing home or living unit were included in the study and investigated with respect to various indicators for dementia (dementia diagnosis in the records and results of cognitive screenings) [24, 54–56]. In these studies, the sample was described, but participants were not selected or assigned based on these indicators. One article is a study protocol; the method to define residents with dementia was not determined, only the measurements that were intended to assess dementia [57]. In 2 publications [58, 59], the method to identify residents with dementia was not reported at all.

**Table 7:** Overview of studies that did not clearly define participants with dementia

Publications	Method of sample determination	Information about dementia diagnosis	Screenings performed to define and describe dementia	Qualification and training of professionals involved
[24]	Every resident on the living unit enclosed in the study and described with respect to dementia-related characteristics	Medical diagnosis is derived from the medical records and coded according to ICD-10.	Presence of dementia diagnosis in the nursing records, DSS (rating according to CDR: mild (CDR 1), severe (CDR 2), very severe (CDR 3)	Screening instruments: Nurses who are familiar with the resident performed the ratings; oral and written instructions were provided by the research team
[54]	Every resident on the living unit included in the study and described with respect to dementia-related characteristics	Not specified	GDS (> 1 beginning dementia, > 3 moderate dementia, > 5 severe dementia), MMSE, CERAD Verbal Fluency Test, CERAD Boston Naming test	Not specified
[55]				
[56]	Every resident on the living unit is enclosed in the study and described with respect to dementia-related characteristics	No information is given on the diagnostic procedures of the existing diagnosis.	Presence of dementia diagnosis in the nursing records, DSS	Screening instruments: Professional nursing staff that were familiar with the resident
[57] (Study protocol)	Every resident on the living unit included in the study and described with respect to dementia-related characteristics	Medical diagnosis is derived from the nursing records.	Presence of dementia diagnosis in the nursing records, DSS, FAST, MMSE as recorded in the nursing records	Screening instruments: Nurses who are familiar with the residents and received training or supervision by a trained study coordinator (NH staff)
[58]	Procedure to identify residents with dementia not reported	Not specified	Not specified	Not specified
[59]				

*CDR* Clinical Dementia Rating, *CERAD* The Consortium to establish a Registry for Alzheimer Disease’s, *DSS* Dementia Screening Scale, *FAST* Functional Assessment Staging, *GDS* Global Deterioration Scale, *MMSE* Mini Mental State Examination

### Reporting about dementia diagnostics within the study

Fifteen articles reporting on studies with a newly assessed diagnosis detailed diagnostic criteria and gave information on the diagnostic process or referred to related articles that described the diagnostic process [22, 23, 48–51, 60–68]. As shown in Table 2, these studies referred to the definition and diagnostic criteria for Alzheimer’s disease and vascular dementia from the ICD, DSM, or NINCDS. Studies that referred to the diagnostic criteria for dementia subtypes reported a more comprehensive diagnostic procedure [22, 60–68] than those reported in studies that did not refer to dementia subtypes. These diagnostic procedures were composed of clinical examinations, interviews, neuropsychological testing and a review of existing diagnostic findings from technical investigations. The articles examined did not specify the number or types of findings available from technical investigations or how missing findings were accommodated.

Three studies did not refer to any definition or diagnostic criteria of dementia, but solely reported the instrument on which the diagnosis was based [69–71]. Two studies conducted prior to 2000 [72, 73], assessed a diagnosis on the basis of the Feighner criteria [74]. Neither of those studies provided any detailed information about the diagnostic process or about which instruments were used to assess the criteria. One pre-1990 study did not report the definition, the diagnostic criteria, or any instrument that was used [75].

The majority of studies in which the diagnosis was newly assessed within the study indicated that a battery of neuropsychological tests was used [22, 23, 48–51, 60–70, 72]. Two of these studies indicated that the final diagnosis was based on the Clinical Dementia Rating (CDR) [69, 70]; in another study, this instrument was used for staging [68].

The compilation of psychological tests differed in the studies with regard to the assessment of cognition, behavior and function. For assessing cognition, the MMSE was the only instrument that was used in all of these studies. Studies using a battery of neuropsychological tests included different tests for cognitive function: the MMSE was combined with the Global Deterioration Scale (GDS), the CDR, the Clock Drawing Test, the Brief Assessment Schedule (BAS-Dem) and the Parkinson Neuropsychometric Dementia Assessment (PANDA). With exception of the PANDA (used for a subsample of residents with Parkinson’s disease) [67] and the CDR (used in cases of severe physical impairment) [66], the studies did not specify whether all of the instruments were administered to each resident or whether any particular instrument was used for residents with certain characteristics. For assessing behavior and function, the researchers also used different instruments, which can be viewed in Tables 2, 3, 4, 5 and 6.

The scope and the rationale of each instrument’s use in the diagnostic process were not clearly documented in most studies. In 10 studies, it was not clear which instruments were essential to the decision about the diagnosis because no cutoff values were reported [22, 23, 60–67]. Moreover, it was not clearly stated which instruments were used for diagnostics and which were used for study purposes only (including the description of the study sample), as an outcome measure, or for validity testing. Five studies reported cutoff values [69–73]; 2 of these studies used the CDR with a cutoff ≥ 1 to assess the dementia diagnosis [69, 70]; 3 studies reported cutoff values for the MMSE and the BAI to stage dementia severity [71–73].

The assessment process for the diagnosis was described briefly in 5 articles: 3 articles reported that the administration of instruments was carried out with the help of interviewers [22, 66, 67], whereas 3 studies reported that the diagnosis was agreed to in a multidisciplinary conference [66, 68, 70]. In all of the studies that assessed a new diagnosis of dementia medical staff with geriatric experience, primarily physicians, performed the diagnostics.

### Reporting about dementia diagnostics prior to the study

Altogether, in 26 articles, the recorded diagnosis was used as an inclusion criterion with or without additional screenings (Tables 3 and 4). Twelve articles reported that existing diagnoses used to determine the sample were performed or confirmed according to ICD [41, 46, 48–51, 76–81]. None of these articles gave more explicit information about the pre-study diagnostic procedure or about whether an existing diagnosis was confirmed by the attending physician. In 14 articles, neither the definition of dementia and/or its diagnostic criteria were reported; in addition, no information was provided about the diagnostic procedure that had been undertaken prior to the study [42–45, 47, 82–90].

In 17 articles, it was reported that in addition to the recorded diagnosis or performed screenings, exclusion criteria were also used in the study [48–51, 53, 76–80, 82, 85–90]. These comprised other neurological or psychiatric diseases that could cause CI.

### Reporting about screening of cognitive impairment

CI was measured in all studies, but with different instruments and cutoff values.

The use of the MMSE was reported in 47 articles and represents the instrument most often used to identify residents with dementia and the stage of its severity. A broad range of cutoff scores for dementia severity was reported for the MMSE. Seven studies referred to an MMSE of < 25 [37, 40, 48–51], 6 studies used a cutoff of ≤ 24 [85–90], 8 articles referred to a cutoff of < 24 to detect mild dementia [76–80, 82, 91, 92], and 4 articles referred to a more conservative cutoff. [39, 41, 83, 93] One article specified a higher cutoff value of ≤ 27 but conducted confirmatory testing with the Geriatric Depression Scale (GDS) for residents with an MMSE between 24 and 27 [84]. One article use a cutoff value of < 27 without additional testing [46]. The studies also used different cutoff values for staging dementia (Table 8).

**Table 8:** MMSE cutoff values for dementia staging^a^

Publication	Severe	Moderate	Mild
[38, 41]	0-9	10-17	18-30^b^
[76–79]	0-9	10-17	18-23
[48–51]	0-9	10-18	19-24
[84]	0-10	11-19	20-27
[37, 40]	0-17	18-23	24-30^b^
[89]	0-9	10-21	> 21^c^
[46]	0-9	10-19	20-26
[92]	0-10	11-17	> 17
[90]	0-9	10-20	> 20^c^

^a^This table only lists publications on studies that used the MMSE for a staging of dementia/CI

^b^Range of values also cover people with no CI/dementia

^c^Participants with a MMSE > 24 were excluded

Thirteen articles did not report a cutoff value for the MMSE but instead reported mean scores for the study sample [22, 23, 60–70].

For the CDR, a consistent cutoff value was reported (≥ 1) in 3 studies [68–70]; 3 articles did not report any cutoff values for the CDR [22, 66, 82]. For the GDS, consistent cutoff values were most often reported. Schumacher et al. and Bär et al. [39, 54, 55] specified mild CI as corresponding to a GDS of 2-3, moderate CI as corresponding to a GDS of 4-5, and severe CI as corresponding to a GDS of 6-7. Pickel et al. [80]. set a cutoff value of > 3 for mild, moderate or severe dementia.

In addition to differences concerning the use of instruments, we also identified differences regarding the professionals who administered the instruments to assess CI. Studies using the assessments to state a dementia diagnosis primarily used physicians. Studies assessing CI without making a statement on the dementia diagnosis used physicians, psychologists, nurses, medical students and students from other health care programs (see Tables 2, 3, 4, 5 and 6). Some studies stated that the assessors were experienced in the field and when nurses were assessors, it was stated that they were familiar with the residents. Training for raters who performed the cognitive assessments was conducted in numerous studies (see Tables 2, 3, 4, 5 and 6); however, the duration of training, training concepts and training evaluation were not specified.

## Discussion

This review reveals a sampling challenge for dementia studies in German nursing homes in health services research: uncertainty related to existing dementia diagnoses, constrained resources and ethical dilemmas concerning accurate diagnostic procedures and the achievement of study validity. When designing a sampling plan, the advantages of convenient and efficient access and ethical considerations related to the residents must be weighed against participant-related validity concerns.

In our review, we found 4 sampling methods to identify residents with dementia:

1. A study diagnosis;
2. A recorded medical diagnosis;
3. A recorded medical diagnosis and additional cognitive screening; or
4. Cognitive or accordant screening.

Each method has to be discussed with regard to its practicability and validity.

A guideline- and criteria-based diagnosis of dementia is the most valid criterion to select study participants when the aim of the study is to target people with dementia. The clear benefits of this method are a thorough assessment of dementia-related conditions and a sound differential diagnosis to ensure that the CI is not caused by conditions other than dementia. Challenges related to this approach are the resources required in terms of time, costs and availability of physicians.

The performance of diagnostics by a member of the research team enhances study validity by ensuring conformity to the criteria. As demonstrated in several studies [22, 23, 48–51, 60–68], criteria conformity can be achieved in the evaluation of a dementia syndrome. In these studies, both subjective and objective information on the patient’s cognitive decline were thoroughly assessed. However, the validity of the dementia subtype diagnosis, as performed in a few of these studies [22, 23, 60–65], was questionable. In particular, during the late stage of dementia, a differentiation between dementia subtypes is difficult and requires the use of disease progression measures such as repetitive cognitive tests. It can be assumed that this information was not readily available for a large number of residents because the implementation of diagnostic measures, other than the collection of subjective information, is infrequent in the German primary care sector. A survey of 23 German primary care physicians regarding the use of diagnostic measures showed that only 3 physicians used neuropsychological tests as a component of their diagnosis [34]. There is also evidence showing that in primary care, imaging techniques and other diagnostic features are seldom used; misclassifications that lead to an overestimation of vascular dementia were frequently discovered in several studies [34, 94, 95].

With respect to the reporting of the diagnostic performance, the studies lacked detailed information. Several studies did not report which instrument was used to assess which criterion, whether normative data were used to determine CI, which cutoffs were used and how missing data were addressed. It is recommended that this information be provided when a dementia diagnosis in the oldest-old population is evaluated [20]. Additionally, information on training for the raters who conducted the cognitive assessments was missing in all of the studies. In our opinion, this is a crucial issue because 1 study showed that even physicians who underwent training in cognitive assessments may still fail to use these diagnostic instruments correctly [34].

However, evaluating a study diagnosis also lacks feasibility when resources are constrained. Imaging diagnostics are critical because nursing home residents are often too frail to transport, and nursing homes are often not affiliated with a clinic. Consequently, appropriate technical devices may not be available. As we can see in the studies that evaluated dementia subtypes, only existing findings were used to conduct a subtype diagnosis. Thus, the diagnosis must be made based on neuropsychological and functional tests, clinical examinations and interviews that are conducted by either geriatric psychiatrists or clinical psychologists; this process requires time, experienced assessors and willing participants. The study with the largest sample size involved 589 residents from twenty nursing homes in a German city [69]. Thus, performing dementia diagnostics within a study seems feasible for a sample size up to 600 people who live in a single city. However, we assume that the evaluation of a dementia diagnosis may have a negative impact on the willingness to participate in a study if the assessment is overly burdening or involves invasive procedures [96]. For example, in 1 study, 20 of 113 eligible residents were unwilling to submit to an MMSE [93]. For the majority of the nursing home population, legal guardians decide whether the resident will participate. In our experience from studies in the field, legal guardians are hesitant to permit participation because studies involve measures that will burden the resident.

The use of a recorded medical diagnosis as an inclusion criteria may be appealing, especially in studies with large samples and with a geographically widely dispersed population. However, the well-documented inaccuracy of recorded dementia diagnoses indicates that in a research situation, this approach must be questioned [6, 30]. If researchers cannot be sure which criteria have been used and how they were measured, we must assume that the diagnosis may not correspond to the established ICD- or DSM-criteria. Consequently, misclassification threatens the internal validity of study results. In experimental trials, diagnostic discrepancies may lead to heterogeneity in the intervention and control group that may have an impact on treatment effects. The inclusion of false-positive participants may explain the failure to show treatment effects, and the exclusion of false-negative participants both prolongs the recruitment process and may cause a selection bias.

The third sampling method that combines a recorded diagnosis with a cognitive screening is more feasible than evaluating a diagnosis following defined criteria and recommended procedures. Cognitive tests are comparatively easy and quick to administer and therefore, their administration can be delegated to nursing-home staff members who are more easily accessible than geriatricians or psychologists, such as nurses or medical students. This method may ensure a more homogenous sample with respect to cognition, but it misses other information about functioning and decline in functionality and cognition. Without this information, it is impossible to verify the diagnosis according to established diagnostic criteria [30, 97]. The risk of including false-positives can be reduced, but not eliminated. In this respect, exclusion criteria are relevant. Several studies reported that residents were excluded when diseases other than dementia explained their cognitive decline. However, the combination of a recorded diagnosis, a cognitive screening and exclusion criteria still does not reduce potential selection bias resulting from systematically missing false negatives. Furthermore, it is necessary to decide what to do when combined inclusion criteria contradict each other. In 1 study, these concerns were addressed. Majic and colleagues report that participants were included with a recorded diagnosis of dementia, an MMSE ≤ 24 and a duration of CI of > 6 months, but diagnostics were performed in case of incongruity [48]. They report elsewhere that additional diagnostics were performed in residents if the nursing home staff suspected dementia, but there was no recorded diagnosis [49]. Other studies did not sufficiently report how they addressed incongruity in the combined inclusion criteria and whether they performed any measures to rule out the outlined problems.

The fourth method that was identified in this review is the use of a single test result. The instruments FAST, DSS and the MMSE were used for this purpose.

The FAST staging procedure is used as a diagnostic measure for dementia and relies on the assessment of functional impairments that are attributable to dementia [98]. This instrument was tested in its original language on a sample that was composed of 16 participants who had different levels of cognitive impairment and numerous possible underlying diseases. The testing was conducted by physicians who were in their postdoctoral training phase; their results revealed excellent values for inter- and intra-rater reliability and concurrent validity [99]. Unfortunately, data on internal consistency and discriminant validity are absent [100] as well as the results from the German version of the FAST. To our knowledge, this instrument has not been evaluated for its sensitivity or specificity when being used to detect dementia. In the reviewed studies, it was administered by health personnel, not physicians. Additionally, there are no studies that compare the accuracy of this instrument depending on if the instrument is administered by physicians versus other health personnel.

The DSS is a 7-item scale that assesses both memory and orientation at the time of assessment [69]. The instrument was originally developed in the German language, tested in 589 nursing home residents, and administered by nurses [69]. It was validated against the MMSE, BAS-DEM and the CDR. Utilizing the CDR as the gold standard, the DSS was able to correctly classify more than 80 % of residents; however, it showed small inaccuracies when compared to the MMSE and BAS-DEM. Because the scale is fast and easy to administer, this approach can be considered to be feasible in health services research, even though its validity must be critically discussed. The scientific literature concordantly states that the assessment of single domains of cognition, especially a single cognitive test, cannot accurately diagnose dementia and should not be used to substitute systematic evaluations, examinations and laboratory tests [101]. Because the DSS does not assess changes in cognitive functions, it is not possible to determine if the present cognitive deficits had declined over the past 6 months. Hence, the DSS will misclassify residents who have acute cognitive deficits that are due to infection or dehydration. The same misclassification can occur in residents who have chronic cognitive impairments due to other diseases.

In our review, the MMSE was the instrument that was most often chosen to define the inclusion criteria. The rationale for using the MMSE in the studies was primarily its degree of popularity and widespread utilization. A meta-analysis on the accuracy of the MMSE confirms this choice: in high prevalence settings, the MMSE shows a sensitivity and specificity of 77 and 90 %, respectively [15]. The notable shortcomings of the MMSE in the context of nursing home research were rarely discussed in the studies, although those shortcomings threatened the internal validity of the study results. The MMSE shows a floor effect in very severe score ranges, people with little formal education, and people with severe language problems. [101] Several items are strongly influenced by age and education, which implies a need to adjust MMSE scores when establishing thresholds. We found a variety of MMSE thresholds for staging dementia in the reviewed studies - a result that was also reported in other reviews [12, 20]. This result may be a consequence of missing normative data for the age group of the oldest-old and standardized age-adjusted cutoff values. The authors of a meta-analysis on dementia screening and case-finding tool validation studies suggest alternatives to the MMSE such as the Mini-Cog [102]. However, the suitability of these screening tools for nursing home research needs to be investigated.

In this respect, an associated question must be discussed in health services research in nursing homes: is a valid diagnosis of dementia necessary to define the study population? If the aim of the study is to prove the benefit of an intervention that should be provided exclusively to residents with dementia, a valid diagnosis is essential to prove the benefit for this population. If the aim of the study is to prove a benefit for residents with cognitive and functional impairments independent of their etiology, a dementia diagnosis is dispensable. The same question must be considered by clinicians because in the absence of disease-modifying treatments, the primary advantage of a diagnosis for the oldest-old population has to be determined to justify resource-intensive diagnostics [20]. The need to perform diagnostics in the nursing home population must also be ethically justified because psychological tests can be significantly burdensome to people with CIs.

We also found 4 articles that aimed to investigate residents who had CI but did not explicitly have dementia. To us, it was unclear why some studies focused on the population of people with CI but not on people with dementia because the authors did not elaborate on the question whether the etiology of CI played a role with respect to their research focus. In 2 publications, the terms “dementia” and “CI” were used interchangeably [39, 40]. If the authors did not explain why they defined their population based on a disease or a symptom and later failed to distinguish between the 2 conditions, one may ask whether the question was sufficiently addressed.

In addition to the variability of case ascertainment strategies in dementia studies, this review mirrors the developments in knowledge, concepts, differentiations and diagnostic criteria related to “dementia” that have occurred during the last 30 years. With each decade of dementia studies in German nursing home research, the diagnostic procedures were refined and reflect methodological innovations. Therefore, the definition and assessment of dementia and CI must be considered against the respective state of knowledge at the time a particular study was conducted. For example, in 1 study from 1993 [72], the Feighner criteria were applied; these criteria had been introduced in the DSM-III and became available in the German language with the German translation of the DSM-III in 1984. On the contrary, another study [75] was published the same year; it is obvious that those researchers could not apply today’s diagnostic criteria because they were not available at the time the study was designed and conducted.

An overview of the advantages and disadvantages of the different case ascertainment strategies is summarized in Table 9.

**Table 9:** Case ascertainment strategies in comparison (summary)

Case ascertainment strategy	Advantages	Disadvantages
Study diagnosis	**▪** The most valid inclusion criterion if recommended diagnostic procedures are followed	**▪** Requires intense resources **▪** Burdens the resident **▪** Is ethically questionable in the nursing home population **▪** Decreases willingness to participate
Recorded diagnosis	**▪** Requires little resources **▪** Easy and quick to assess **▪** No burden for the resident **▪** Increases willingness to participate	**▪** Validity of the diagnosis cannot be assured **▪** Residents without a recorded diagnosis are systematically excluded **▪** Potential inclusion of false-positives **▪** Differential diagnosis is often missing
Recorded diagnosis and screening result	**▪** Requires little resources **▪** Easy and quick to assess **▪** No burden for the resident if proxy-ratings are used **▪** Increases willingness to participate if the resident is not burdened with assessment procedures	**▪** Validity of the diagnosis cannot thoroughly be assured, but with the help of screening results false-positives can be detected and verified **▪** Residents without a recorded diagnosis are systematically excluded, unless residents with a probable diagnosis are also screened and a new diagnosis is evaluated **▪** Validity of the recorded diagnosis cannot be assured **▪** Differential diagnosis is often missing
Screening result	**▪** Requires little resources **▪** Easy and quick to assess **▪** No burden for the resident if proxy-ratings are used **▪** Increases willingness to participate if the resident is not burdened with assessment procedures	**▪** The declaration of the existence of a dementia is not entirely possible **▪** Enables the selection of residents that are homogenous with regard to the screened condition but cannot prevent heterogeneity of other conditions

### Strengths and limitations

The problem of inconsistent management of methodological challenges in dementia research has already been recognized and has led to an initiative on how to improve the reporting of these challenges in clinical studies [103]. However, studies of the outlined problem of inconsistent case ascertainment strategies in nursing home research have been lacking to date. We consider this as a prerequisite for improving dementia research in the nursing home sector and outline this as the major strength of this study.

The generalizability of the displayed results is constrained to one country (Germany). However, we assume that the outlined problem can also be demonstrated in other countries, but this must be proven in a later review. Perhaps in countries in which physicians are employed in nursing homes and adherence to diagnostic standards can be guaranteed, the reliance on a recorded diagnosis can be received less critically.

As discussed, the reporting of diagnostic procedures was partially insufficient; in particular, different publications from a single study provided varied information about their case ascertainment. In some studies, it was not clear whether the assessments were used exclusively for study purposes, for diagnosis, or both. In particular, the scope of assessments with respect to behavior and function was not described clearly, making it unclear whether these instruments were also used for diagnostic procedures. In an effort to rule out this lack of clarity, we unsuccessfully attempted to contact the authors. The short reporting made it also impossible to assess the quality of diagnostic procedures or the associated risk of bias.

Because of the descriptive objective of the review, it has not been registered in a review register.

## Conclusions

Considering the findings of our review, we suggest the following principles to improve the validity and comparability of study results on dementia in nursing homes.

Investigators must clearly distinguish whether their research addresses residents with CIs or residents with dementia with an appropriate etiology. If residents with dementia are addressed, it should be in accordance with the study aim. Studies that address the population of nursing home residents with dementia should report in detail which method of case ascertainment was used and should discuss the limitations associated with each method. In our opinion, a criterion-based diagnosis is essential when a study addresses explicitly residents with dementia. If residents are included in studies based on diagnoses that do not conform to established criteria, the results from different studies are not comparable. To ensure that a diagnosis conforms to criteria, clinicians who are members of the research team should evaluate a diagnosis during the research project. When financial or human capacities are constrained, one should consider alternative methods to a face-to-face consultation by a geriatric physician, psychologist or psychiatrist. Several methods utilizing an expert panel are described for the diagnosis of dementia [104]. The method by Magaziner et al. [30] appears promising for research purposes: 2 clinicians decide whether dementia is present based on the resident’s history and neuropsychological tests performed by lay evaluators. Compared to direct clinical evaluation, agreement was satisfactory (76 %). Another promising approach is a nurse-administered diagnosis. Studies showed a high agreement between a nurse-administered diagnosis and a multidisciplinary team diagnosis in determining mild CI in primary care [105]; in addition, there is agreement between a Memory Clinic diagnosis [106] and a moderate agreement with the ICD-10 diagnosis (ibd.). To our knowledge, a nurse-administered diagnostic procedure for dementia in nursing home residents has not yet been developed and tested.

Regardless of which method is used to evaluate a diagnosis of dementia within a study, reporting requires details regarding the domains that are tested, the rationale behind instrument use for each domain examined, the cutoff values set for each domain and the management of missing data. Transparency in reporting missing data is needed because approaches for adequately managing these gaps can differ and produce varying effect estimates [103].

If a dementia diagnosis is considered as dispensable or a criteria-based diagnostic procedure cannot be applied due to constrained resources, the study should not target the dementia population, but instead clearly state which population they are addressing.

## Ethics approval and consent to participate

Not applicable.

## Consent for publication

Not applicable.

## Availability of data and materials

All data are contained within the manuscript and its supplementary files.

## Additional file

Additional file 1:
**Table S1.** PRISMA checklist. (DOCX 63 kb) Additional file 2:
**Table S2.** Search strategy in MEDLINE. **Table S3.** Search strategy in CINAHL. (DOCX 17 kb)

## Abbreviations

AD: Alzheimer’s disease
ADL: activites of daily living
AES: Apathy Evaluation Scale
AKT: Alters-Konzentrationstest
B-ADL: Bayer-Activities of Daily Living Scale
BAGI: Bielefelder Autobiografisches Gedächtnisinventar
BAI: Brief Assessment Interview
BAS-Dem: Brief Assessment Schedule
Behave-AD: Behavioral Pathology in Alzheimer’s Disease
BPRS: Brief Psychiatric Rating Scale
CDR: Clinical Dementia Rating
CERAD: The Consortium to Establish a Registry for Alzheimer’s Disease
CI: Cognitive Impairment
DSM: Diagnostic Statistical Manual
DSS: Dementia Screening Scale
E-ADL: Erlangen Test for Activities of Daily Living
FAST: Functional Assessment Staging
GDS: Global Deterioration Scale
HDRS 17: Hamilton Depression Scale 17
IADL: Instrumental Activities of daily living
ICD: International Classification of Diseases
interRAI LTCF: International Resident Assessment Instrument Long Term Care Facility
MCI: Mild Cognitive Impairment
MMSE: Mini Mental State Examination
NINCDS: National Institute of Neurological and Communicative Disorders and Stroke
NOSGER: Nurses’ Observation Scale
NPI: Neuropsychiatric Inventory
PANDA: Parkinson Neuropsychometric Dementia Assessment
PRISMA: Preferred Reporting Items for Systematic Reviews and Meta-Analyses
PSMS: Physical Self Maintenance Scale
SIDAM: Structured Interview for the Diagnosis of Alzheimer’s Dementia, Multi-infarct dementia and dementias of other etiology
