# Supplementary material for: People with dementia in nursing home research: a methodological review of the definition and identification of the study population
Source: BMC Geriatr. 2016 Apr 5;16:78. doi: 10.1186/s12877-016-0249-7 (PMC4823911; doi:10.1186/s12877-016-0249-7)
Supplement: Additional file 2: — Table S2. Search strategy in MEDLINE. Table S3. Search strategy in CINAHL. (DOCX 17 kb) [file 12877_2016_249_MOESM2_ESM.docx]

## Appendix

## Table A 1: Search strategy in MEDLINE [PubMed]

Conducted on 08/12/2015

| **Nr.** | **Search syntax** | **Results** |
| --- | --- | --- |
| 1 | Dementia [MeSH] | 126273 |
| 2 | Alzheimer* [tiab] | 103297 |
| 3 | Demen* [tiab] | 80422 |
| 4 | Cognition [Mesh] | 116989 |
| 5 | Cognition disorders [Mesh] | 67178 |
| 6 | MCI [tiab] | 11832 |
| **7** | **1 OR 2 OR 3 OR 4 OR 5 OR 6** | **337684** |
| 8 | Nursing home [Mesh] | 33113 |
| 9 | nursing home [tiab] | 17152 |
| 10 | Homes for the Aged [MeSH] | 11572 |
| 11 | Residential Facilities [MeSH] | 44569 |
| 12 | Resident* [tiab] | 129722 |
| 13 | Elderly home*[tiab] | 187 |
| **14** | **8 OR 9 OR 10 OR 11 OR 12 OR 13** | **165188** |
| 15 | German* | 1572615 |
| 16 | German [tiab] | 50079 |
| **17** | **15 OR 16** | **1572615** |
| **18** | **7 AND 14 AND 17** | **584** |
|  | ((((German [tiab]) OR German*)) AND (((((((Demen*[tiab]) OR Alzheimer*[tiab]) OR Dementia[MeSH])) OR MCI [tiab]) OR Cognition disorders [Mesh]) OR Cognition [Mesh])) AND ((((((nursing home[Mesh]) OR nursing home[tiab]) OR Homes for the Aged[MeSH]) OR Residential Facilities[MeSH]) OR Resident*[tiab]) OR Elderly home*[tiab]) | |

## Table A 2: Search strategy in CINAHL [EBSCO]

| **Nr.** | **Search syntax** | **Results** |
| --- | --- | --- |
| 1 | Alzheimer’s Disease | 17400 |
| 2 | AB Alzheimer* | 7648 |
| 3 | Dementia | 30416 |
| 4 | AB Demen* | 14978 |
| 5 | Cognition | 36450 |
| 6 | Cognition disorders | 13626 |
| 7 | AB MCI | 1117 |
| **8** | **1 OR 2 OR 3 OR 4 OR 5 OR 6 OR 7** | **72080** |
| 9 | MH "Nursing Homes” | 15745 |
| 10 | TI Homes for the aged OR AB Homes for the aged | 1642 |
| 11 | (MH "Residential Facilities") | 2849 |
| 12 | TI Resident* OR AB Resident* | 30465 |
| 13 | TI elderly home* OR AB elderly home* | 2403 |
| **14** | **9 OR 10 OR 11 OR 12 OR 13** | **45798** |
| 15 | German* | 16383 |
| 16 | TI German* OR AB German* | 9045 |
| **17** | **15 OR 16** | **16383** |
| **18** | **8 AND 14 AND 17** | **66** |

Conducted on 08/12/2015
